# Overexpression of ICAM-1 Predicts Poor Survival in High-Grade Serous Ovarian Carcinoma: A Study Based on TCGA and GEO Databases and Tissue Microarray

**DOI:** 10.1155/2019/2867372

**Published:** 2019-06-13

**Authors:** Shasha Wang, Can Yin, Ying Zhang, Lu Zhang, Lin Tao, Weihua Liang, Lijuan Pang, Ruiting Fu, Yusong Ding, Feng Li, Wei Jia

**Affiliations:** ^1^Department of Pathology, Shihezi University School of Medicine, Xinjiang 832002, China; ^2^Department of Obstetrics and Gynecology, The First Affiliated Hospital School of Medicine, Shihezi University, Xinjiang 832002, China; ^3^Department of Preventive Medicine, School of Medicine, Shihezi University, Xinjiang 832002, China; ^4^Department of Pathology and Medical Research Center, Beijing Chaoyang Hospital, Capital Medical University, Beijing 100020, China

## Abstract

Intercellular cell adhesion molecule-1 (ICAM-1), an important adhesion molecule in the immunoglobulin superfamily, is expressed on many cell types. Recent studies have identified ICAM-1 as a potential oncogene that promotes the development of epithelial ovarian cancer (EOC); it was also found to be associated with poor survival. However, the clinical significance of its expression in high-grade serous ovarian carcinoma (HGSOC) is unclear. Thus, this study aimed to investigate the significance of ICAM-1 expression in HGSOC. Data on* ICAM1* expression and mutations in serous ovarian carcinoma (SOC) were obtained from the Cancer Genome Atlas (TCGA), and* ICAM1* mRNA expression data in HGSOC were obtained from the Gene Expression Omnibus (GEO) database. ICAM-1 expression was evaluated by immunohistochemistry in HGSOC and normal fallopian tube tissues microarray. In TCGA data, amplification/mutation of* ICAM1* was identified in 12% of serous ovarian carcinoma samples, and overexpression of* ICAM1* mRNA predicted reduced overall survival in SOC. From TCGA and GEO data, SOC patients with* ICAM1* mRNA overexpression treated with chemotherapeutic drugs that contained taxol or taxol and platin together had significantly reduced progression-free survival. According to GEO data,* ICAM1 *mRNA expression was found significantly higher in HGSOC than in control samples. In our study, ICAM-1 overexpression was observed in 63.1% (65/103) of HGSOCs. As a prognostic biomarker, overexpression of ICAM-1 predicted reduced recurrence-free and overall survival and is an independent risk factor for poor prognosis. These findings suggest that overexpression of ICAM-I is an independent indicator of poor prognosis for HGSOC and that it can serve as an effective clinical prognostic biomarker for this disease.

## 1. Introduction

Ovarian carcinoma is the most lethal gynecological malignancy worldwide [1]. High-grade serous ovarian carcinoma (HGSOC), the most common subtype, accounts for 75–80% of epithelial ovarian carcinomas (EOCs) [2]. Due to a lack of effective biomarkers for early detection and screening, approximately 75% of patients with HGSOC are diagnosed at advanced stages, and the 5-year overall survival rate (20–30%) has not significantly improved over the last 20–30 years [3, 4]. Therefore, exploring novel biomarkers involved in HGSOC progression and prognosis would contribute to early diagnosis and provide therapeutic targets.

Intercellular cell adhesion molecule-1 (ICAM-1), also known as CD54, is an important adhesion molecule of the immunoglobulin superfamily. As a transmembrane leukocyte and endothelial cell protein, ICAM-1 enhances tumor cell adherence to endothelial cells [5]. An increasing number of studies have elucidated that ICAM-1-mediated interactions with macrophages enhance the immunosuppressive function of human mesenchymal stem cells [6], and ICAM-1 overexpression enhances rhinovirus replication in monocytic cells [7]. Moreover, this molecule regulates the immune response and has a role in tumorigenesis. In liver cancer, ICAM-1 promotes tumor formation and metastasis, and its overexpression is correlated with poor prognosis [8]. Research had reported that ICAM-1 was found to be highly expressed in EOC, promoting tumorigenesis and metastasis [9]. However, its expression in HGSOC tissues has not been confirmed; accordingly, its role in the development and prognosis of this disease remains ambiguous and thus requires further investigation.

Here, the TCGA database was used to examine the relationship between* ICAM1* amplification and mRNA expression in SOC, and the correlation between* ICAM1* mRNA expression and prognosis was analyzed. We further examined expression in HGSOC and control samples using the GEO database and assessed ICAM-1 protein expression in HGSOC and normal fallopian tube tissues by immunohistochemistry. Furthermore, the prognostic significance of ICAM-1 expression, based on clinicopathological characteristics and HGSOC patient survival, was evaluated.

## 2. Materials and Methods 

### 2.1. TCGA and GEO Analysis

An in silico analysis using the TCGA dataset was performed as previously reported [10]. The TCGA SOC dataset was selected using the cBioPortal online platform [11].* ICAM1* amplification was queried using the OncoPrint function. The plot function illustrated the correlation between copy number variance (CNV)/mutation and mRNA expression. All statistical analyses were performed automatically by the cBioPortal platform and a P-value < 0.05 and Q-value < 0.05 were accepted as statistically significant. Gene Expression Omnibus (GEO) datasets including GSE18520, GSE105437, and GSE10971 were downloaded from the GEO database [http://www.ncbi.nlm.nih.gov/geo/], and all gene expression data were determined using only the HG-U133 Plus 2.0 Affymetrix microarray platforms. Specifically,* ICAM1* expression was determined using 202637_s_at probe. Ninety-eight high-grade serous ovarian carcinomas, 24 normal ovarian surface epithelium tissues, and 12 normal fallopian tube tissues were used for analysis. The TCGA and GEO SOC datasets were selected using the Kaplan–Meier Plotter online platform [http://kmplot.com/analysis/].* ICAM1* mRNA expression was determined using the 202637_s_at probe (with the same 202637_s_at probe used for the GEO database so that comparisons could be made between datasets). The best cut-off value for* ICAM1* mRNA expression was autoselected by this online platform. The numbers of available samples from SOC patients treated with chemotherapeutic drugs that contained taxol, platin, or taxol and platin together for this online progression-free survival analysis were 715, 1259, and 698, respectively.

For survival analysis of TCGA HGSOC patients, high-throughput sequencing expression data (period ending January 28, 2016) for* ICAM1* from 295 available samples from patients with SOC were downloaded using R software (R 3.4.2). The “RTCGAToolbox” library was used for this analysis. The median* ICAM1* mRNA expression value was used as the cut-off to divide the samples into high-expression and low-expression groups. The median, minimum, and maximum* ICAM1* mRNA expression values were 30.81, 1.03, and 347.59, respectively.

### 2.2. Patient Information

In total, 103 and 41 formalin-fixed, paraffin-embedded HGSOC and normal fallopian tube tissue specimens (one from each patient), respectively, were obtained from the Department of Pathology of the First Affiliated Hospital of Shihezi University School of Medicine. The collection of specimens was approved and supervised by the Ethics Committee of the First Affiliated Hospital of Shihezi University School of Medicine. Clinical data from patients with HGSOC, including age, presence of ascites, clinical stage, chemotherapy response, recurrence-free survival, and overall survival, were collected from the medical records room of the First Affiliated Hospital of Shihezi University and from the electronic medical record system. Tumor stages were assessed in accordance with the International Federation of Gynecology and Obstetrics (FIGO) staging system, which refers to the 2014 FIGO surgical staging criteria for ovarian, fallopian, and peritoneal cancer [12]. Recurrence-free survival was defined as the time from surgery to relapse or until the study endpoint. overall survival was calculated as the time from surgery to death or until the endpoint of the study, which was December 5th, 2017. No patients in this study received chemotherapy or radiotherapy before surgery, and the majority underwent chemotherapy followed by surgical debulking of tumor mass, as summarized in Table 1. For each tumor sample, hematoxylin and eosin (HE)-stained slides were re-reviewed independently by two experienced pathologists to confirm the final diagnosis. Neither pathologist had any knowledge of clinical information or ICAM-1 expression status.

### 2.3. Tissue Microarray Construction

Tissue microarrays (TMAs) were created in accordance with established methods [13]. One representative paraffin-embedded tissue block was selected from each patient, and the selected area, with representative morphology, was labeled on HE slides. Two cores were then taken from the block. Each was reviewed based on HE staining to ensure that tumor cells were present in at least 70% of the core.

### 2.4. ICAM-1 Immunohistochemical Examination

Immunohistochemical staining was performed on 5-*μ*m sections that were deparaffinized and rehydrated. Heat-induced antigen retrieval was performed at 95°C for 30 min in citrate buffer (pH 6.0). A monoclonal mouse antihuman antibody against ICAM-1 (clone: 4915S, 1:50 dilution; Cell Signaling Technology, Burlingame, CA) was used to immunostain the sections. Antibody-conjugated peroxidase activity was detected using the EnVision System (Dako, Carpinteria, CA) and the 3,3′-diaminobenzidine peroxidase substrate kit (Dako). The sections were then counterstained with hematoxylin, dehydrated, and mounted. PBS (phosphate buffer saline), instead of primary antibody, was used as a negative control. ICAM-1 protein levels were assessed by microscopically examining stained TMA slides. Membrane ICAM-1 immunoreactivity was scored semiquantitatively by dividing the number of positively stained tumor cells by the total number of tumor cells. The immunohistochemical scoring system used here has been widely established [14]. The positive cells (range, 0–100%) were multiplied by the staining intensity score (1, buff; 2, brownish yellow; 3, brown). Scores greater than or equal to 100 (the best cut-off value) were considered overexpression, whereas scores less than 100 were considered low expression.

### 2.5. Statistical Analysis

The relationships between ICAM-1 protein expression and clinicopathological characteristics and the associations between ICAM-1 protein levels within the normal fallopian tube and HGSOC samples were analyzed by chi-squared tests. Nonparametric test was used to determine the differences in* ICAM1* mRNA/protein expression between normal fallopian tube/ovarian surface epithelium and HGSOC groups. Survival analysis was performed using the Kaplan–Meier method and the log-rank test was used to compare between the two groups. Univariate and multivariate analyses using the Cox regression model were conducted to determine the independent significance of relevant clinical covariates. All tests were two-sided.* P *< 0.05 was considered significant and all analyses were performed using Statistical Product and Service Solutions (SPSS) software (version 20.0; SPSS, Chicago, IL).

## 3. Results

### 3.1. ICAM1 Expression in TCGA and GEO HGSOC Patients

**Figure 1:**
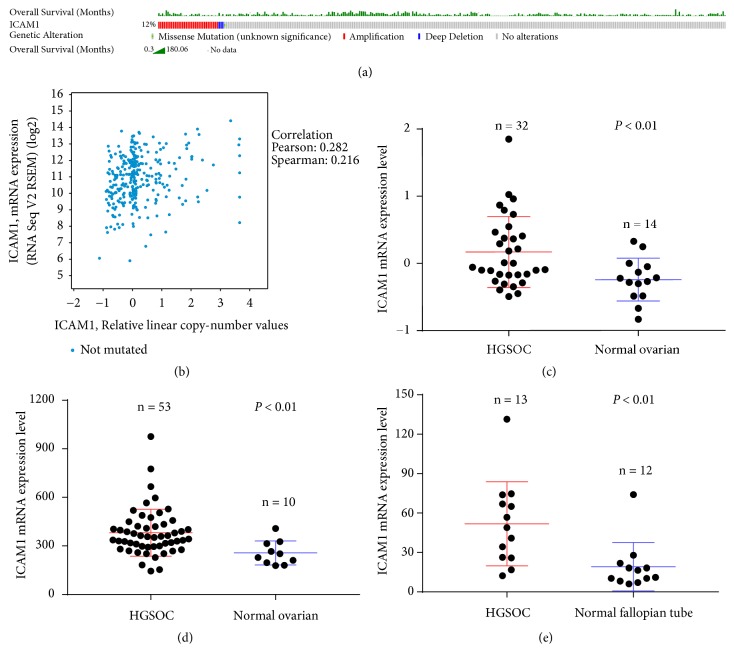
Analysis of* ICAM1* expression based on TCGA, Provisional (a, b) and GEO datasets (c, d, e). (a) Gene alterations in* ICAM1* in serous ovarian carcinoma. (b)* ICAM1* mRNA levels were consistent with gene amplification. Functional plotting of the corresponding mRNA levels based on the TCGA database. ((c), (d), (e) correspond to GSE105437, GSE18520, GSE10971, respectively)* ICAM1* mRNA levels were higher in high-grade serous ovarian carcinoma samples than in normal samples.

**Figure 2:**
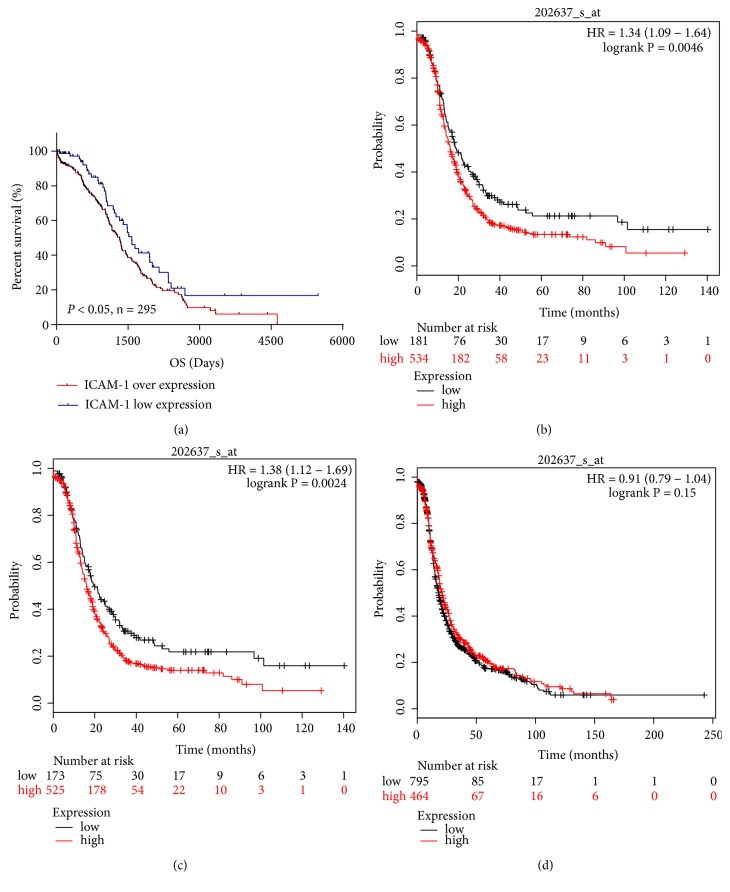
Analysis of the relationship between* ICAM1* mRNA expression and prognosis of patients with serous ovarian carcinoma based on TCGA (a) and TCGA/GEO (b, c, d) datasets. (a) Patients with* ICAM1 *mRNA overexpression had shorter overall survival than those with low* ICAM1* mRNA expression (P < 0.05). (b, c) Serous ovarian carcinoma (SOC) patients with* ICAM1 *mRNA overexpression treated with chemotherapeutic drugs that contained taxol or taxol and platin together had shorter progression-free survival (*P* < 0.01 for both). (d) There was no significant difference in progression-free survival for the SOC patient group that was treated with chemotherapeutic drugs which contained platin (*P* > 0.05).

There were 311 and 307 patient SOC samples in the TCGA for which mutation/CNV and mRNA expression data were available, respectively (TCGA, Provisional).* ICAM1 *amplification/mutation was observed in 12% of cases (Figure 1(a)). Amplification (10%) was the predominant alteration in* ICAM1*. Functional plotting of* ICAM1* data revealed that amplification was associated with increased mRNA expression (Figure 1(b)). GEO data analysis showed that* ICAM1* mRNA expression was significantly higher in HGSOC cases than in normal samples (all* P* < 0.01; Figures 1(c), 1(d), and 1(e)). Among the downloaded TCGA data, the 295 patient SOC samples included clinical survival information. Kaplan–Meier survival analysis indicated that patients with* ICAM1* mRNA overexpression had significantly reduced overall survival compared to those with low* ICAM1* expression (*P* < 0.05; Figure 2(a)). Based on Kaplan–Meier Plotter analysis of TCGA and GEO data, SOC patients with* ICAM1* mRNA overexpression treated with chemotherapeutic drugs that contained taxol or taxol and platin together had significantly reduced progression-free survival compared to that in SOC patients with low* ICAM1 *expression (*P* < 0.01; Figures 2(b) and 2(c)). However, this group that was treated with chemotherapeutic drugs containing platin had no significant difference in progression-free survival (*P* > 0.05; Figure 2(d)).

### 3.2. Clinicopathological Characteristics of HGSOC

The clinicopathological characteristics of patients with HGSOC recruited for this study are summarized in Table 1. The median age was 53 years (range, 25–75). Stage I, II, III, and IV tumors were observed in 12 (11.6%), 20 (19.4%), 24 (23.3%), and 15 (14.6%) cases, respectively, whereas tumor stage was unknown in 32 (31.1%) cases. Moreover, 44 (42.7%) individuals had ascites, 16 (15.5%) did not, and 43 (41.8%) cases were unknown; 103 patients underwent chemotherapy after surgery: 36 (35.0%) were sensitive to this treatment, 20 (19.4%) were resistant, and 47 (45.6%) were unknown.

### 3.3. ICAM-1 Expression Is Significantly Correlated with HGSOC

**Figure 3:**
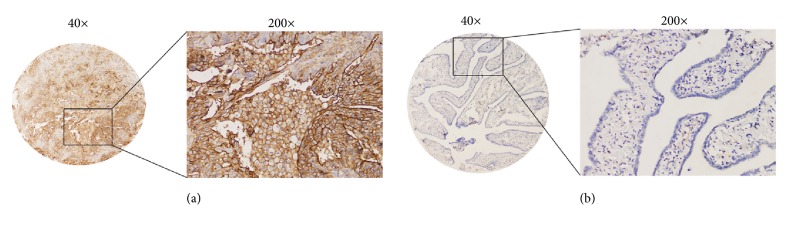
Immunohistochemical staining for ICAM-1 in serous ovarian carcinoma and normal fallopian tube microarray tissues (on the same array and slide). (a) Overexpression of cytomembrane ICAM-1 in high-grade serous ovarian carcinoma. (b) Absence of ICAM-1 staining in the normal fallopian tube tissue. Original magnification: ×40 and ×200, respectively.

Immunohistochemical staining for ICAM-1 expression was performed in 103 HGSOC and 41 normal fallopian tube tissue samples (Figures 3(a) and 3(b)). ICAM-1-positive staining was predominantly localized to the cytomembrane, and all HGSOC specimens were positive for this marker. Expression varied markedly between cancerous and noncancerous tissues. ICAM-1 overexpression was detected in 65 (63.1%) of 103 HGSOC cases and 11 (26.8%) of 41 fallopian tube specimens; this difference was significant (*P* < 0.001; Table 2). Expression of ICAM-1 was further analyzed in HGSOC (I-II stage) vs. normal fallopian tubes, being statistically significant (*P* < 0.05, Supplementary Figure 1).

### 3.4. Association between ICAM-1 Expression and Clinicopathological Characteristics

Based on association analysis, the expression of ICAM-1 was significantly higher in advanced-stage tumors than in early-stage advanced-stage tumors (*P = *0.038; Table 1) and was also elevated in chemotherapy-resistant cases compared to levels in chemotherapy-sensitive cases (*P = *0.041; Table 1).

### 3.5. ICAM-1 Is Correlated with Decreased Survival among Patients with HGSOC

**Figure 4:**
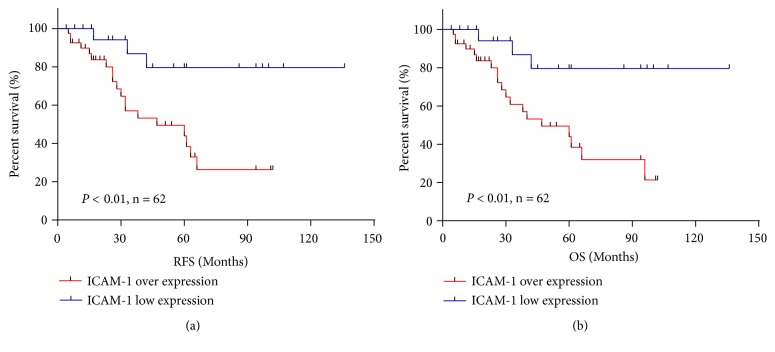
Prognostic significance of ICAM-1 expression in high-grade serous ovarian carcinoma. Patients with ICAM-1 overexpression had shorter recurrence-free survival (a) and overall survival (b) than those with low ICAM-1 expression (*P* < 0.01 for both).

Among the 62 patients with HGSOC for which follow-up information was available, the median recurrence-free survival and overall survival were both 47 months. At the endpoint, 22 patients had died, and 44 were alive. Kaplan–Meier survival analysis indicated that patients with ICAM-1 overexpression had significantly reduced recurrence-free survival and overall survival compared to those with low expression (*P* < 0.01 for both; Table 3, Figures 4(a) and 4(b)). Based on univariate analysis using the Cox regression model, ICAM-1 overexpression was considered a strong prognostic factor of poor overall survival (*P *= 0.011; Table 4). In addition, advanced stage (*P *= 0.001) and chemotherapy response (*P *= 0.045) were associated with shorter overall survival. For multivariate Cox regression analysis, only variables that were statistically significant based on univariate Cox regression analysis were included, and the results identified stage (*P* = 0.042) as an independent prognostic factor (Table 4).

## 4. Discussion

ICAM-1 is expressed on many cell types and upregulated through the activation of multiple pathways including PKC*α*-p38-SP-1, JAK, PI3K, AKT, and NF-*κ*B [15, 16]. Its role in ovarian cancer has gradually become a research hotspot, but few studies have been conducted on HGSOC, the most common histological type of ovarian cancer.

The 4th edition of the WHO classification classifies ovarian serous carcinoma as HGSOC and low-grade serous ovarian carcinoma (LGSOC) [17]. The current consensus is that the origins of LGSOC and HGSOC are different. LGSOC develops slowly from the ovarian epithelium and serous ovarian tumors and has a good prognosis, whereas HGSOC develops from the fallopian tube epithelium with high invasiveness and poor prognosis [18]. Previous studies demonstrated that* ICAM-1* mRNA is highly expressed in EOC tissues, cell lines, and serum [9, 19], but without further subhistological type analysis. Analysis of TCGA datasets revealed that* ICAM1 *amplification occurs in 10% of SOC samples, and the mRNA expression was also found to be increased with amplification. Furthermore, using high-throughput* ICAM1* mRNA sequencing data and clinical survival analysis of TCGA data, high* ICAM1* mRNA expression was found to be significantly associated with poor prognosis in SOC. Combined Kaplan–Meier Plotter analysis of TCGA and GEO data revealed that SOC patients with* ICAM1* mRNA overexpression treated with chemotherapeutic drugs that contained taxol or taxol and platin together had significantly reduced progression-free survival compared to that in SOC patients with low* ICAM1* expression. However, there was no significant difference in progression-free survival for the group that was treated with chemotherapeutic drugs that contained platin. This suggests that patients with high ICAM-1 expression might be resistant to paclitaxel. Our study also showed that high ICAM1 expression is associated with chemoresistance. We further speculate that high expression of ICAM1, leading to poor prognosis in HGSOC patients, might be related to chemotherapy resistance, but this needs to be tested experimentally. In the TCGA and GEO databases, the subtypes of SOC were not available; however, as HGSOC accounts for the vast majority of SOCs, we could still find a trend for HGSOC from those data.

Furthermore, analysis using GEO data (with the same 202637_s_at probe of TCGA database) revealed that* ICAM1 *mRNA expression was significantly higher in HGSOC tissue than in normal ovarian or fallopian tube tissue. These results are consistent with those of Cai et al. [19] who proved that* ICAM1* mRNA is highly expressed in EOC and that high expression is significantly associated with shortened overall survival. These data indicate that high* ICAM1* mRNA expression might be associated with HGSOC progression. Hence, we hypothesize that* ICAM1* amplification and high mRNA expression might lead to increased protein expression in HGSOC.

We further detected ICAM-1 protein expression in 103 HGSOC tissues and found that it was significantly higher than that in 41 fallopian tube tissues, implying that high expression might play a role in HGSOC development. We further analyzed the expression of ICAM-1 in HGSOC (I-II stages) and normal fallopian tubes. ICAM-1 expression was higher in patients with asymptomatic stages (HGSOC, I-II stages) than in normal persons, which may indicate that ICAM-1 overexpression may lead to advances in early detection of HGSOC. Our results are consistent with those of previous studies [20]. The current consensus is that HGSOC originates from the fallopian tube epithelium [18]. Our study found ICAM-1 overexpression in 26.8% of normal fallopian tube samples (11/41). This finding might suggest that ICAM-1 expression in normal fallopian tube tissues affects normal physiological cellular function to promote malignant transformation. These data support the notion that* ICAM1* could be a potential oncogene in HGSOC.

Many studies have revealed that high ICAM-1 expression promotes the occurrence, metastasis, and poor outcome of many kinds of carcinoma such as cervical [21], hepatocellular [8], gastric [20], and prostate cancer [22] and that it might be induced by NF-*κ*B- and AP-1-mediated pathways [23]. However, it has also been reported that ICAM-1 is expressed at low levels in some carcinomas, even in ovarian carcinoma. Arnold et al. showed downregulated ICAM-1 in various histological types of ovarian adenocarcinoma [24]; perhaps, due to the different mechanisms to detect different histological types of EOC, the mismatch of various histological types might have led to biased results. Groote et al. also demonstrated that ICAM-1 is expressed at low levels in ovarian carcinoma cells and that ICAM-1 overexpression reduces ovarian carcinoma A2780 cell growth in the absence of immune cells [25], which suggested that the immune microenvironment of tumors affects the function of ICAM-1. Therefore, the biological behavior of ICAM-1 in HGSOC requires an in-depth study.

In the current study, we found that advanced tumor stage and chemotherapy tolerance were associated with high ICAM-1 expression in HGSOC and that ICAM-1 overexpression is associated with an increased risk of death for HGSOC. Our study demonstrated that patients with ICAM-1 overexpression have significantly shorter recurrence-free and overall survival. These results are consistent with those of previous studies, which proved that ICAM-1 is highly expressed in EOC and is significantly associated with shortened overall survival [19]. Taken together, these data indicate that high ICAM-1 expression is associated with poor prognosis in patients with HGSOC and could be used as a biomarker to predict prognosis. To our knowledge, this is the first study to identify the association between ICAM-1 and HGSOC prognosis.

## 5. Conclusions

In conclusion, we demonstrated that ICAM-1 overexpression occurs in 63.1% of HGSOCs and that high expression is correlated with advanced stage and chemotherapy resistance. More importantly, ICAM-1 overexpression was associated with an increased risk of death for this disease. We further speculate that high* ICAM1 *expression, leading to poor prognosis in HGSOC patients, might be related to chemotherapy resistance; however, further experimental testing is required to confirm this. These findings indicate that* ICAM1*, a candidate oncogene, is involved in the development of HGSOC and could be used as a prognostic biomarker for this disease. The role of* ICAM1* as a potential oncogene in HGSOC requires further experimental validation.

## Figures and Tables

**Table 1:** Association between ICAM-1 expression and clinicopathological parameters in patients with high-grade serous ovarian carcinoma.

Characteristics	n = 103	ICAM-1 Expression		X^2^	*P*
		Over-expression (%)	Low expression (%)		
Age					
≤50	42	22 (52.4)	20 (47.6)		
>50	61	43 (70.5)	18 (29.5)	3.504	0.061
Stage					
Early (I-II)	31	15 (48.4)	16 (51.6)		
Advanced (III-IV)	40	29 (72.5)	11 (27.5)	4.309	0.038**∗**
unknown	32				
Ascites					
No	16	9 (56.2)	7 (43.8)		
Yes	44	29 (65.9)	15 (34.1)	0.471	0.492
unknown	43				
Chemotherapy resistance					
No	36	21 (58.3)	15 (41.7)		
Yes	20	17 (85.0)	3 (15.0)	4.192	0.041**∗**
unknown	47				

**Table 2:** Expression of ICAM-1 in high-grade serous ovarian carcinoma and fallopian tube.

Group	n	ICAM-1 Expression		X^2^	*P*
		Over-expression(%)	Low expression (%)		
high-grade serous ovarian carcinoma	103	65 (63.1)	38 (36.9)	15.486	< 0.001**∗∗∗**
fallopian tube	41	11 (26.8)	30 (73.2)		

**Table 3:** Univariate analysis for recurrence free survival and overall survival.

Variables	Median RFS (months)	*P*	Median OS (months)	*P*
Age		0.869		0.897
>50	132		132	
≤50	63		63	
Stage		< 0.001**∗∗∗**		< 0.001**∗∗∗**
Early (I-II)	132		132	
Advanced (III-IV)	38		38	
Ascites		0.946		0.878
Yes	61		61	
No	38		38	
Chemotherapy resistance		0.032**∗**		0.032**∗**
Yes	32		33	
No	undefined		undefined	
ICAM-1 expression		0.005**∗∗**		0.005**∗∗**
Overexpression	47		47	
Low expression	undefined		undefined	

Abbreviations. RFS: recurrence free survival; OS: overall survival.

**Table 4:** COX regression analysis of risk factors in patients with high-grade serous ovarian carcinoma.

Characteristics	Univariate analysis		Multivariate analysis	
	HR (95%CI)	*P*	HR (95%CI)	*P*
ICAM-1 expression				
Low expression	1		1	
Overexpression	4.924 (1.448,16.744)	0.011**∗**	1.847 (0.331,10.322)	0.484**∗**
Age				
>50	1			
≤50	0.939 (0.379,2.325)	0.897		
Stage				
Early (I-II)	1		1	
Advanced (III-IV)	11.296 (2.596,49.151)	0.001**∗∗**	11.230 (1.090,115.705)	0.042**∗**
Ascites				
No	1			
Yes	1.094 (0.349,3.432)	0.878		
Chemotherapy resistance				
No	1		1	
Yes	3.587 (1.027,12.537)	0.045**∗**	1.612 (0.482,5.387)	0.438

Abbreviations. HR: hazard ratio; CI: confidence interval.

## Data Availability

The data used to support the findings of this study are included within the article.

## Supplementary Materials

Supplementary Materials
* Supplementary Figure 1*: analysis of ICAM-1 expression in high-grade serous ovarian carcinoma (I-II stage) and normal fallopian tube microarray tissues. ICAM-1 levels were higher in high-grade serous ovarian carcinoma (I-II stages) samples than in normal samples (*P* < 0.05).
