# Supplementary material for: Overexpression of ICAM-1 Predicts Poor Survival in High-Grade Serous Ovarian Carcinoma: A Study Based on TCGA and GEO Databases and Tissue Microarray
Source: Biomed Res Int. 2019 Jun 13;2019:2867372. doi: 10.1155/2019/2867372 (PMC6595389; doi:10.1155/2019/2867372)
Supplement: Supplementary Materials — Supplementary Figure 1: analysis of ICAM-1 expression in high-grade serous ovarian carcinoma (I-II stage) and normal fallopian tube microarray tissues. ICAM-1 levels were higher in high-grade serous ovarian carcinoma (I-II stages) samples than in normal samples (P < 0.05). [file 2867372.f1.pdf]

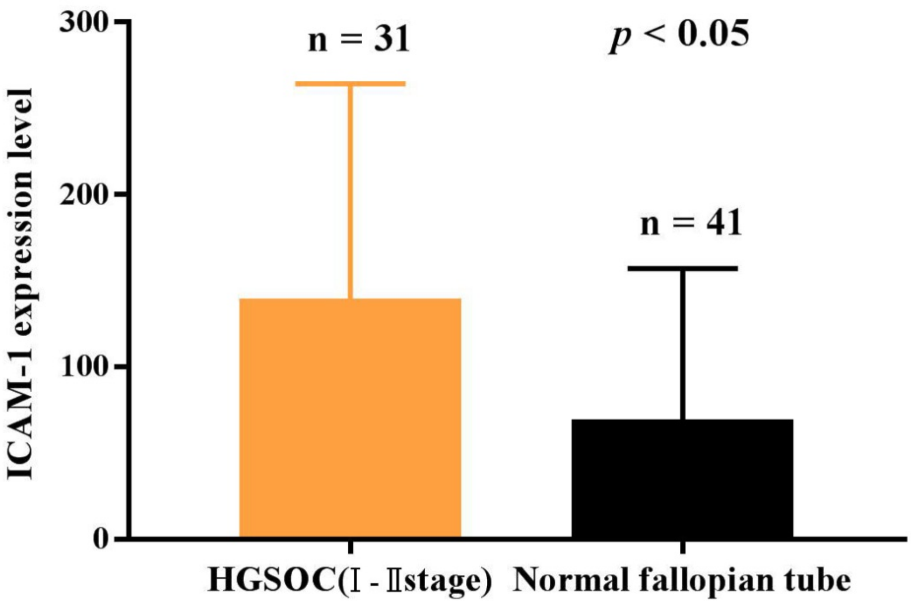

Supplementary Figure 1: Analysis of ICAM-1 expression in high-grade serous ovarian carcinoma (Ⅰ-Ⅱstage) and normal fallopian tube microarray tissues. ICAM-1 levels were higher in high-grade serous ovarian carcinoma (Ⅰ-Ⅱstage) samples than in normal samples ($P < 0 .05$).
